# Editorial: Advanced Biomaterials and Systems Releasing Bioactive Agents for Precise Tissue Regeneration 

**DOI:** 10.3389/fbioe.2021.763685

**Published:** 2021-09-08

**Authors:** Rui Guo, Gang Wu

**Affiliations:** ^1^ Key Laboratory of Biomaterials of Guangdong Higher Education Institutes, Guangdong Provincial Engineering and Technological Research Centre for Drug Carrier Development, Department of Biomedical Engineering, Jinan University, Guangzhou, China; ^2^ Department of Oral and Maxillofacial Surgery/Pathology, Amsterdam UMC and Academic Center for Dentistry Amsterdam (ACTA), Vrije Universiteit Amsterdam (VU), Amsterdam Movement Science (AMS), Amsterdam, Netherlands; ^3^ Department of Oral Cell Biology, Academic Centre for Dentistry Amsterdam (ACTA), University of Amsterdam and Vrije Universiteit Amsterdam, Amsterdam, Netherlands

**Keywords:** biomaterials, tissue engineering, bioactive agent, bone regeneration, modification

To provide a scientific forum for the researchers in this field, we started this research topic—*Advanced Biomaterials and Systems Releasing Bioactive Agents for Precise Tissue Regeneration* in February 2020. Until now, there are two mini reviews, four reviews and 11 original research articles are published with about 100 authors involved. We hereby sincerely thank all the efforts and contributions of these excellent researchers to this successful research topic.

Bone regeneration is still one of the hottest topic in the field of tissue engineering with nine articles (one mini review, one review and seven original researches) published in this direction. These authors reported the functionalization of biomaterials with various types of bioactive agents, such as icaritin (naturally derived small molecule) and Ginsenoside Rg1, BMP-2 (recombinant proteineous growth factor), histatin-1 (naturally derived peptide), four chemical elements, such as silicon and copper, to significantly improve the bio-functions of biomaterials, such as osteoconductivity, osteoinductivity, and anti-bacterial properties. The biomaterials involve metallic implants and ceramic bone-defect-filling materials without or with the incorporation of organic phase to improve their properties, such as osteoconductivity and slow release of bioactive agents. In addition to the studies reporting the development of novel regenerative materials, Liu et al., also reported the incorporation of ultrasmall superparamagnetic iron oxide nanoparticles in bone grafts, which could facilitate the non-invasively monitor the biodegradation and remodeling of bone grafts after implantation. Furthermore, Li et al. also reported a simplified and effective method to create an experimental murine periodontitis model, which could be used to study the pathological mechanisms of periodontitis and developing potential periodontal tissue regeneration strategies. Apart from the original research articles, two reviews were also also published, which focused on smart hydrogel-based bioink/scaffolds for bone tissue regeneration and bioactive modification strategy to promote osseointegration in osteoporotic microenvironment, respectively.

Apart from bone tissue engineering, the authors also made excellent reports and reviews to summarize recent advances, provide monitoring method and novel reconstructive materials and alternative treatment options for diverse diseases, such as macular degeneration, gastric cancer, bile duct restoration, urethra, periphery nerve injury, and coronary artery disease. In addition to the above disease-targeting publications, there is still one review focusing on the synthesis, modification, characterization and medical applications of AuNPs.

In summary, this research topic covers many recent significant advances in bioactive agents-functionalized biomaterials with diversely targeting diseases, which make a good contribution to the progress of advanced biomaterials for precise tissue regeneration.

